# Cost-effectiveness of multidisciplinary management of Tinnitus at a specialized Tinnitus centre

**DOI:** 10.1186/1472-6963-9-29

**Published:** 2009-02-11

**Authors:** Rilana Cima, Manuela Joore, Iris Maes, Dyon Scheyen, Amr El Refaie, David M Baguley, Johan WS Vlaeyen, Lucien Anteunis

**Affiliations:** 1 Clinical Psychological Science, Maastricht University, Maastricht, The Netherlands; 2 Department of Clinical Epidemiology and Medical Technology Assessment, University Hospital Maastricht, Maastricht, The Netherlands; 3 Rehabilitation Foundation Limburg, Hoensbroek, The Netherlands; 4 Access and Communication Studies, Bristol University, Bristol, UK; 5 Departments of Otolaryngology and Audiology, Addenbrooke's Hospital, Hills Road, Cambridge, UK; 6 Department of Psychology, University of Leuven, Leuven, Belgium; 7 Department of Otorhinolaryngology & Head and Neck Surgery, University Hospital Maastricht, Maastricht, The Netherlands

## Abstract

**Background:**

Tinnitus is a common chronic health condition that affects 10% to 20% of the general population. Among severe sufferers it causes disability in various areas. As a result of the tinnitus, quality of life is often impaired. At present there is no cure or uniformly effective treatment, leading to fragmentized and costly tinnitus care. Evidence suggests that a comprehensive multidisciplinary approach in treating tinnitus is effective. The main objective of this study is to examine the effectiveness, costs, and cost-effectiveness of a comprehensive treatment provided by a specialized tinnitus center versus usual care. This paper describes the study protocol.

**Methods/Design:**

In a randomized controlled clinical trial 198 tinnitus patients will be randomly assigned to a specialized tinnitus care group or a usual care group. Adult tinnitus sufferers referred to the audiological centre are eligible. Included patients will be followed for 12 months. Primary outcome measure is generic quality of life (measured with the Health Utilities Index Mark III). Secondary outcomes are severity of tinnitus, general distress, tinnitus cognitions, tinnitus specific fear, and costs. Based on health state utility outcome data the number of patients to include is 198. Economic evaluation will be performed from a societal perspective.

**Discussion:**

This is, to our knowledge, the first randomized controlled trial that evaluates a comprehensive treatment of tinnitus and includes a full economic evaluation from a societal perspective. If this intervention proves to be effective and cost-effective, implementation of this intervention is considered and anticipated.

**Trial Registration:**

The trial has been registered at ClinicalTrial.gov. The trial registration number is NCT00733044

## Background

### Problem definition

#### The disease

Subjective tinnitus is the involuntary perception of the concept of a sound without the presence of an external source. It is a chronic condition that is highly prevalent, especially among hearing impaired individuals. Studies show a prevalence of 10% to 20% in the general population [1,2] and among hearing impaired individuals prevalence has been estimated at 75% to 80% [3]. Of the Dutch population at least 2 million individuals suffer from some form of tinnitus, 340.000 individuals indicate to hear the tinnitus continuously and 60.000 individuals claim to be severely impaired in their daily activities [4]. Among severe sufferers it causes disability associated with severe affective problems, major declines in concentration, sleeping difficulties, hypersensitivity to sounds and problems in (re-)directing attention. The combination of these complaints makes them feel exhausted and frustrated resulting in diminished quality of life [5-9]. Tinnitus is known to occur as a concomitant of almost all the dysfunctions that involve the human auditory system [1], and it is postulated that the aetiology of tinnitus is diverse and that different activation circumstances can be present [10]. Little is known about the pathophysiology and there is no known drug or curative therapy at present [11] though considerable research effort has been expended in this regard.

#### The health care problem

In many cases tinnitus sufferers are referred to different caregivers in a non-standardized way, and often receive insufficient and sometimes inappropriate treatment. This may comprise prescribing a drug that is not proven to be effective, or informing the patients that not much can be done to improve the situation. Especially in those individuals suffering from a moderate to severe tinnitus, incorrect information and delay of appropriate treatment is expected to increase psychological strain, aggravation of tinnitus severity, and prolongation of the referral trajectory [11]. Since tinnitus sufferers seek help in various areas of health care without receiving appropriate treatment, they are financially burdening the system superfluously. In absence of a proven cure or uniformly effective treatment, tinnitus care is often fragmentised and costly [12].

#### Usual Care

As for most health problems in the Dutch population, the general practitioner (GP) is the initial professional to consult for patients with tinnitus. In most cases, within six months after onset of subjective tinnitus the individual consults his GP, but one quarter of the respondents wait several years until they seek help [4]. In the official Dutch GP patient information letter on tinnitus (URL: ), it is stated that there is not much that can be done to alleviate complaints. Another frequently consulted specialist is the ENT physician. Treatment possibilities include removal of cerumen, medication, and audiological rehabilitation. Generally, the effects of these treatments are disappointing.

#### Motivation and relevance for the chosen intervention

A recent study by El Refaie et al (2004) shows that functional and social handicap in tinnitus sufferers is significantly reduced, and quality of life improves significantly, as a result of attendance at a specialised tinnitus clinic. Specialised clinics for chronic disorders such as tinnitus and chronic pain have been proven to be most effective in treatment [13]. Similarities between tinnitus and chronic pain in terms of cognitive and behavioural mechanisms [14] have been suggested recently and a similar treatment could be effective for the tinnitus population. As in chronic pain, multidisciplinary specialised treatment is more effective in ameliorating severe tinnitus complaints than monodisciplinary treatments. A retrospective pilot study, by the applicants of this proposal, in the Tinnitus Centre Limburg (TCL) shows significant improvements in 71% of the patients (N = 41). Intrusiveness of the tinnitus ameliorates in 85% of the subjects and 78% experiences improvement in emotional distress caused by the tinnitus.

### Objective

The objective of this study is to examine the effectiveness, costs and cost-effectiveness of a comprehensive multidisciplinary treatment provided by a specialised tinnitus centre. Treatment is based on a stepped care approach, tailored to individual needs, with key elements from cognitive behavioural therapy, education, relaxation techniques, attention diversion, exposure in daily live situations, and tinnitus retraining therapy.

The following research questions were formulated:

1. What are the effects on generic quality of life of comprehensive specialized tinnitus care as provided by a specialised tinnitus centre, as compared to usual care?

2. What are the effects on health, in terms of negative affect, tinnitus beliefs, fear of the tinnitus, and tinnitus annoyance, of comprehensive specialized tinnitus care as provided by a specialised tinnitus centre, as compared to usual care?

3. What are the costs to health care and to society of treatment provided by a specialised tinnitus centre in the Dutch health care system as compared to usual care?

4. What is the cost-effectiveness of treatment provided by a specialised tinnitus centre in the Dutch health care system as compared to usual care?

## Methods/design

### Design

**Figure 1:**
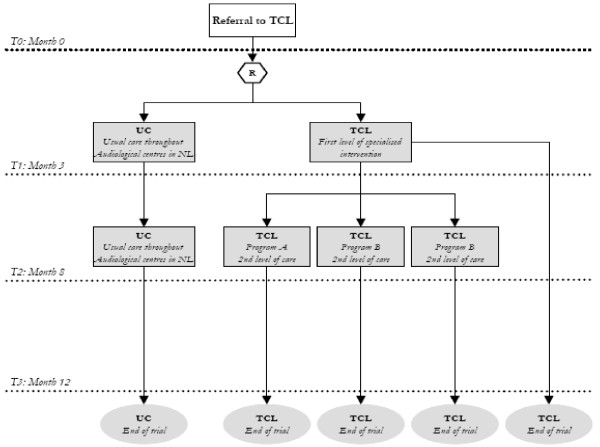
**Flowchart levels of care within randomized controlled clinical trial in Tinnitus Centre Limburg**.

A randomised controlled clinical trial will be performed, with 2 conditions (Fig. 1). Patients will be assigned to a Usual Care (UC) Control condition or a Tinnitus Centre Limburg care (TCL) condition. Both treatment conditions (UC and TCL) will be provided by the Audiological Centre Hoensbroeck. Measures will be taken for blinding patients to treatment assignment.

For assessing the cost-effectiveness, the TCL care group will be compared only to the UC group and not to other treatment programs. The analysis will be performed from a societal perspective.

### Participants

The study population consists of tinnitus sufferers referred to the TCL, with subjective tinnitus complaints, aged 18 years and older. Exclusion criterion is not being able to write and read in Dutch.

Inclusion of patients started on September 1^st ^2007 and will proceed until the targeted number of patients is reached, for a maximum of 18 months. It is expected that enough patients will be referred to the TCL during this period to reach the necessary number as was calculated by power-analysis.

### Sample size calculation and feasibility of recruitment

After attending a specialised tinnitus clinic a change of 0.065 in health state utility as measured with the SF-6D has been observed [9]. To detect this difference (assuming a two-sided significance level = 0.05, power = 80%, standard deviation of the difference = 0.15), 86 persons per group are needed. Taking into account 15% loss to follow up, the required sample size is 99 persons per group (198 persons in total).

Approximately 400 individuals suffering from tinnitus apply to TCL yearly. We expect this number to stay stable or even increase in the coming years. Therefore it is expected that it will not be necessary to actively recruit patients for this trial.

### Patient allocation and randomization

Research information in written format and a declaration of willingness to participate in the trial will be sent to all new patients of the Tinnitus Centre Limburg that are registered with subjective tinnitus complaints. If a patient declares that he or she is willing to participate in the study they will be invited for the baseline measurement. This face-to-face contact will be used to determine whether the patients understood the information correctly and a written informed consent will be obtained. If they agree, a hearing test will be performed to determine the level of hearing loss and the patients are asked to fill in the tinnitus questionnaire (TQ) [15] to determine the severity of the tinnitus. Based on the Fletcher Index and the scores on the TQ the patient will be randomly assigned to one of the treatment groups. Since treatment depends on tinnitus severity and the severity of hearing loss it will be important that these two prognostic factors are equally presented in the UC group and the TCL group.

Treatment allocation will be achieved by block randomisation (four blocks; A, B, C & D) to ensure equal and balanced groups. A randomization list was generated using randomization software. An equal number of patients will be allocated to the TCL group and the UC group. Patients with a score equal to or less than 46 on the TQ and a Fletcher Index below 60 dB will be allocated to block A. Patients with a score equal too or less than 46 on the TQ and a Fletcher Index equal to or above 60 dB will be allocated to block B. Patients with a score above 46 on the TQ and a Fletcher Index below 60 dB will be allocated to block C. Finally, patients with a score equal to or below 46 on the TQ and a Fletcher Index above or equal to 60 dB will be allocated to block D. The randomization procedure will be performed by an independent person at a location outside TCL.

### Intervention

The intervention consists of comprehensive tinnitus management provided by a specialized tinnitus centre in the health care system. The tinnitus centre offers care following a stepped-care approach with two levels (see Fig. 1). Stepped care is a framework for organizing health services based on patients' needs, with a gradual increase in the intensity of the care at each level [16].

The first level of intervention consists of a basic multidisciplinary intervention for all patients referred to TCL. This multidisciplinary intervention consists of audiological diagnostics (Table 1) and intervention, a tinnitus educational group session and an individual consult with a clinical psychologist. For patients with mild complaints this basic multidisciplinary intervention is expected to suffice.

**Table 1:** Audiological diagnostics and intervention in first level

**Audiological diagnostics and intervention**
1. Pure tone and speech audiometry
2. Uncomfortable Loudness Level measurement
3. Tympanometry: including stapedial reflexes
4. Hearing aid check and optimisation (if present)
5. Tinnitus analyses: Pitch Mask Frequency and Masking level
6. Tinnitus anamnesis using structured questionnaire
7. Individual consult by clinical physicist in audiology (60 minutes)

For patients with moderate to severe complaints a second level of intervention exists. This second level of intervention consists of combinations of the following therapies: Cognitive Behavioural Therapy (CBT), Attention Diversion (AD) by means of movement therapy to build up a more positive mind-body relationship, exposure techniques, and Relaxation Therapy (RT). The programs are preferably offered in group format. The group treatments are based on the theoretical framework of the fear-avoidance model proposed by Lethem and colleagues [17], refined by Vlaeyen and Linton [18], and a cognitive behavioural model by Kröner-Herwig [8] explaining factors in the development and maintenance of chronic tinnitus. Based on existing knowledge in chronic pain management, Folmer et al [14] formulated treatment strategies possibly effective for patients suffering from chronic tinnitus, or as they put it, chronic phantom "pain". The authors conclude that severity of depression, anxiety and insomnia is highly correlated with the severity of the tinnitus, similar to chronic pain. They suggest that techniques and strategies effective in treating chronic pain disorder, might be useful in treating tinnitus as well. These include: stress management techniques (including relaxation therapy) to reduce physiological reactivity, cognitive-behavioural techniques to reduce catastrophising cognitions and reduce avoidance behaviours and exposure to fear-eliciting stimuli to adjust for estimations of the tinnitus sound. The treatment under investigation consists of three main programs namely; program A for patients suffering from tinnitus on a moderate to severe level, program B for severe tinnitus complaints, and program C for the severely hearing impaired suffering from tinnitus. All programs are based on the principals stated above. Depending on severity of complaints and hearing loss, group treatment is more intense and tailored to individual needs. In a review from Andersson and Lyttkens [19] it was concluded that offering cognitive behavioural coping techniques in combination with relaxation exercises received the most empirical support.

### Usual Care

Usual care consists of a standardized version of the treatment that is currently applied in peripheral audiological centres throughout the Netherlands for tinnitus patients. A telephone survey was conducted amongst all audiological centres (n = 28) in the Netherlands. The results of this survey determined the content of the usual care treatment protocol in the current study. The treatment consists of audiological diagnostics and intervention and, if necessary, one or more consultations with a social worker with a maximum of ten one hour sessions.

### Outcomes and instruments

Primary outcome measure:

· Generic quality of life, as measured with the Health Utilities Index Mark 3 (HUI3) [20]

Secondary outcome measures:

· Anxiety and depression as measured with the Hospital Anxiety and depression Scale (HADS) [21];

· Tinnitus related disability and handicap, as measured with the Tinnitus Handicap Inventory (THI) [22];

· Tinnitus annoyance and severity, as measured with the Tinnitus Questionnaire (TQ) [15];

· Tinnitus-related fear was assessed by the Fear of Tinnitus Questionnaire (FTQ). This novel 17-item questionnaire is based on the Tampa scale for Kinesiophobia [23] and the Pain Anxiety Symptoms Scale [24];

· Dysfunctional beliefs and/or cognitions regarding the tinnitus, as measured with the Tinnitus Coping and Cognition list (TCCL). The TCCL is a recent adaptation of the Pain Coping and Cognition Questionnaire [25];

· Catastrophic (mis)interpretations of tinnitus, as measured with the Tinnitus Catastrophising Scale (TCS). The TCS is a recent adaptation of the Pain Catastrophising Questionnaire [26];

· Costs, as measured with a retrospective cost questionnaire.

### Data collection

Measurement of the HUI3, TQ, THI, HADS, FTQ, TCCL, TCS and a cost questionnaire will take place at four moments during a 12 month period. At baseline (T0) the questionnaires will be completed at the audiological centre in the presence of a research assistant. Three (T1), eight (T2) and twelve (T3) months after baseline the patient will be able to complete the questionnaires at home through the internet. Login codes will be sent to their home address two weeks in advance. If patients are incapable of completing the questionnaire through the internet, a paper version will be provided. Non-responders will receive a telephone call as a reminder to complete the questionnaires. If they do not wish to further participate in the study, the reasons for their withdrawal will be recorded.

### Data-analysis

Intention-to-treat analysis will be performed, including all patients that were originally enrolled in the study, irrespective of whether they completed the therapy. To test the differences between the conditions, mixed multilevel regression analyses will be used with a hierarchical backward elimination method. The analysis will be carried out for the POST treatment assessments (after level 1 and level 2 respectively) and follow-up data of the outcome variables. The independent variables are: PRE measurements of the dependent variable, treatment condition, treatment centre, sociodemographics, tinnitus-related variables, and the interaction variable PRE-measurement*treatment. The treatment condition always remains in the regression model, but the other independent variables will be added to increase the power of the analysis and are subsequently eliminated to keep only the significant ones. At each step of the analysis, tests will be done to check for high co-linearity (VIF>10) and/or outliers (Cook's Distance (Cook D)) and Studentised Residual (Sresid)). If Cook D > 1 the case will be removed from the analysis. If Sresid < -3 or > 3, the case will be removed providing that Cook D of this case is considerably higher than from the other cases. By looking at plots of the relationship between each independent variable and the dependent variable, a possible curvilinear relationship is excluded. The prediction errors will be also checked for normality (zresid). For each dependent variable, the initial regression model includes all independent variables and interaction mentioned above. Non-significant interactions (p > .05) will be deleted from the model. Next, non-significant (p > .10, two-tailed) predictors will be deleted one by one, except the treatment factor that always remains in the model. If a significant interaction is found, the treatment effect will be evaluated within strata defined by the covariate interacting with the treatment.

Based on the results of the intention-to-treat analysis, additional per protocol analysis will be performed, incorporating only those patients that completed the therapy. The same analyses as according to the intention-to-treat principle will be performed with respect to the primary outcomes.

### Economic Evaluation

A cost-effectiveness analysis will be performed from a societal perspective. Since both effects on costs and generic health-related quality of life are to be expected, the method of economic evaluation is a cost-utility analysis. The primary effect parameter is generic health-related quality of life, measured in quality adjusted life years (QALYs). The time horizon of the study is one year, identical to the duration of the follow up in the clinical study. The immediate treatment effects (measurements at 3 and 6 months) and short-term treatment effects (measurement at 12 months) are observed in this study. It is not possible to observe long-term treatment effects (longer than12 months), since the duration of the study is limited to three years. Discounting is not relevant given the one-year time horizon. Sampling uncertainty surrounding the incremental cost-utility ratio will be estimated by non-parametric bootstrapping. Confidence intervals for the incremental cost-utility ratio will be calculated from the bootstrap results. The implications of sampling uncertainty on decision uncertainty (the probability specialised tinnitus care provided in a specialised tinnitus centre is more cost-effective than usual care) will be quantified using the cost-effectiveness acceptability curve. Sensitivity analyses will be used to show the impact of variation in non-stochastic input parameters on the incremental cost-utility ratio, such as discount rate, unit prices, and design issues. The impact of variability on the incremental cost-utility ratio arising from diversity and heterogeneity in the patient population will be examined in subgroup analyses. Costs in the analysis include direct health care costs (medical costs for prevention, diagnostics, therapy, rehabilitation and care), direct non-health care costs (travel costs) and indirect costs (productivity loss). Resource use will be measured using the case-record forms and 3 monthly retrospective cost-questionnaires. In the cost questionnaires the PRODISQ modules will be used to estimate productivity loss [27]. When available, the standard unit costs from the Dutch Manual for Cost Analysis [28] will be used. Resource use for which no standard unit costs are available will be valued using integral cost calculations. Costs from productivity loss will be quantified using the friction cost method, as recommended in the Netherlands [28].

### Ethical considerations

Patients will be informed verbally and in written format about the research project before they sign the informed consent form. Participants can retreat from the study at any moment. This will have no influence on their further treatment.

The study protocol has been reviewed and approved by the Medical Ethical Board of the Rehabilitation Foundation Limburg.

The scientific merits of the study protocol have been reviewed in the consecutive phases of research funding process by the independent reviewers of the funding organization ZonMw, the Netherlands Organization for Health Research and Development.

### Funding

A grant was obtained in a competitive application process of the efficacy research program, round 2006, of the Netherlands Organization for Health and Development ZonMw.

## Discussion

### Potential strengths of the study protocol

#### Design

To our knowledge this is the first randomized controlled clinical trial that evaluates a comprehensive multidisciplinary treatment of tinnitus versus care as usual. A particular strength is the randomization procedure, in which allocation is concealed. Randomization is done at the patient level and stratified on degree of hearing impairment and tinnitus severity. This procedure is performed by an external independent person.

#### Sample size

To our knowledge this is the first study evaluating a comprehensive multidisciplinary treatment of tinnitus that includes a large sample size. At least 198 patients with tinnitus will be included in the study. As a result most statistical procedures will be robust against violations of assumptions that have to do with normality.

#### Recruitment strategy

In this randomized controlled trial every recruited patient experiences tinnitus to be one in three of their major complaints. Since tinnitus does not have to be the primary problem it is ascertained that different severity levels of tinnitus will be evaluated in this study.

#### Competence of health care professionals

Every discipline is trained to perform the intervention in a uniform way. To get insight into their actual performance, every professional is required to register all activities during all treatment-related activities during patient visits. This registration will be used to search for factors related to the intervention that might influence effectiveness.

### Potential limitations of the study protocol

#### Intervention

There is no uniform way of treating tinnitus in the audiological centres in the Netherlands. In order to model usual care treatment, a telephone survey was conducted amongst all audiological centres. This implicates that the currently implemented form of usual care is standardized, whereas in real practice clinical variation in treatment is expected.

#### Randomization approach

Randomization on patient level could lead to contamination, and bias the results of this study. However, the influence of contamination is minimised since patients in the usual care group have no access to the intervention offered by TCL-specialists and vice versa. Nevertheless it is possible that specialists that provide the usual care treatment, are more attentive to the usual care group than would be expected if treatment was provided in an independent centre. As a result our findings may be conservative.

## Conclusion

This study will provide information on whether a comprehensive, multidisciplinary treatment is more effective and efficient care for tinnitus patients. The results will also show whether the specialised treatment improves quality of life and patient satisfaction. If the intervention is proven to be effective, implementation of the intervention is considered and anticipated. First results are not expected before the beginning of 2010.

## Competing interests

The authors declare that they have no competing interests.

## Authors' contributions

All authors have read and approved the final manuscript. RC, the researcher and first author of this manuscript, is involved in the development of the experimental intervention, the design of the study, data collection, statistical analysis and performed the power calculation. MJ is involved in the design of the study, wrote the economic evaluation part of the study protocol, supervises the economic evaluation, the planning and the project and is involved in revising the article for important intellectual content. IM was helpful in writing this manuscript and is involved in the design of the study, the data collection and the statistical analysis for the economic evaluation part of the study. DS is involved in the development of the experimental intervention, the design of the study and the instruction of the health care professionals regarding this intervention. AER and DB gave advice regarding the study protocol and are involved in revising the study protocol for important intellectual content. JV and LA supervise the project and are involved in the development of the experimental intervention, the design of the study and revising the study protocol for important intellectual content.

## Pre-publication history

The pre-publication history for this paper can be accessed here:


